# The Endothelium Abridges Insulin Resistance to Premature Aging

**DOI:** 10.1161/JAHA.113.000262

**Published:** 2013-06-21

**Authors:** Angelo Avogaro, Saula Vigili de Kreutzenberg, Massimo Federici, Gian Paolo Fadini

**Affiliations:** 1 Department of Medicine, University of Padova, Italy (A.A., S.V.K., G.P.F.); 2 Venetian Institute of Molecular Medicine, Padova, Italy (A.A., G.P.F.); 3 Department of Systems Medicine, University of Rome Tor Vergata, Rome, Italy (M.F.); 4 Center for Atherosclerosis, Policlinico Tor Vergata, Rome, Italy (M.F.)

**Keywords:** cardiovascular disease, diabetes, stem cells

## Introduction

Although there are different mechanistic theories for aging,^1^ endothelial dysfunction (ED) is a rather neglected player in the aging process. A maladaptive insulin/IGF‐1‐like signaling (IIS) has a remarkable importance in proaging mechanisms, and insulin has direct effects on ED.^2^ Therefore, we assume that the endothelium plays a key role in mediating the aging process in the presence of maladaptive insulin signaling. This latter condition leads to insulin resistance and affects several aspects involved in premature aging, such as body composition, mitochondrial activity, and endocrine function. The present review highlights key mediators and mechanisms responsible for the link between endothelial dysfunction, insulin resistance and aging. In particular, we discuss the sirtuin‐1 system, the p66Shc pathway, telomeres, and their interrelationships with endothelial damage and repair.

## Endothelial Dysfunction: Consequence and Predictor of Insulin Resistance and Metabolic Diseases

ED is considered a common ground of type 2 diabetes (T2DM) and cardiovascular disease (CVD).^3^ The ability of insulin to recruit nutritive capillaries that receive little or no blood flow in fasting conditions is a component of insulin‐mediated glucose uptake. Therefore, in the capillary and arteriolar beds, which are in intimate contact with metabolically active insulin‐sensitive tissues, ED leads to insulin resistance and T2DM.^4^ The relationship between ED and glucose tolerance is experimentally and clinically solid. Insulin‐mediated glucose uptake is lower in *eNOS*^−/−^ mice than in wild‐type C57Bl/6 mice,^5^ as insulin induces vasodilatation in the skeletal muscle via increasing NO.^6^ In addition, genetic manipulation of the insulin signaling pathway leads to ED and insulin resistance.^7–9^ Stehouwer and colleagues proposed that insulin resistance syndrome (or metabolic syndrome) components can be viewed as diverse consequences of ED.^10^ More specifically, these authors hypothesized that approximately 40% of insulin‐mediated glucose uptake by skeletal muscle can be attributed to capillary recruitment; according to this hypothesis, microvascular dysfunction not only precedes and predicts the development of T2DM but also constitutes one of the links between insulin resistance and hypertension in metabolic syndrome.^11^ Consistent with this view, elevated levels of endothelial activation biomarkers such as ICAM‐1 and other adhesion molecules predict incident diabetes.^12–13^ Based on these data, it is reasonable that ED predicts insulin resistance and diabetes that, in turn, anticipate and accelerate the aging process. Aging is also typically associated with impaired glucose tolerance, mainly because of a decline in insulin action.^14–15^ In contrast, high insulin sensitivity is linked to longevity, and parental longevity is inversely correlated with the risk of diabetes.^16–17^ A remarkable body of data in support of this mutual relationship is also available and detailed in other reviews.^18^ From these works, it emerges that (1) insulin resistance can lead to ED, (2) ED can contribute to insulin resistance, and (3) both insulin resistance and ED accelerate aging.

## Aging in the Vascular System

The life expectancy of diabetic individuals is estimated to be lower than that of the general population by 9.1 years among males and 6.7 years among females.^19^ The identification of longevity‐associated genes in the vascular endothelium, along with evidence of their abnormal expression in the context of ED and insulin resistance, suggests that in the aging process, endothelial dysfunction and insulin resistance are intimately linked.

### The Sirtuin System in Metabolism and Endothelial Function

Caloric restriction (CR) is the most consistent experimental model of increased life span and protection from aging‐associated diseases. Evidences indicate that the positive effects that CR exerts on diabetes and CVD are mediated by sirtuins. A decade ago, the silent information regulator 2 (*SIR2*) gene was shown to extend the life span of budding yeast. Since then, much has been understood about sirtuin biology,^20^ and although their effect on life span has been disputed, new data confirmed that sirtuin action is relevant for the improvement of metabolic disorders.^21–22^ Importantly, animal and human studies have shown that CR prevents diabetes and protects from CVD. The mammalian sirtuin (Sirt)–1 is highly expressed in endothelial cells and controls functions that are critical to suppressing the development of atherosclerosis.^23^ A series of experimental studies have shown that Sirt1 plays a role in improving the function of endothelial cells (Table).

**Table 1.** Reported Relationships Between Sirt‐1 and Endothelial Function

Authors	Model	Mechanisms	Readout	Mediator
Kim et al^24^	BAECs, HUVECs, HepG2s	Regulation of endothelial sprout and angiogenic activity	Postnatal vessel development	Methyl‐CpG‐binding protein MeCP2
Mattagajasingh et al^23^	Rat aortic rings	eNOS	Increased NO production	Deacetylation through lysines 496 and 506 in the calmodulin‐binding domain of eNOS
Ota et al^25^	HUVECs	Deacetylation of p53	Altered expression of PAI‐1 and eNOS	Impaired EGF‐induced activation of MAPK
Potente et al^26^	Mixed SV/129×C57Bl/6 mouse endothelial cells	Altered expression of genes encoding for Flt1, CXCR4, Pdgfß, angiopoietin‐like 2, Mmp14, and EphB2	Sprouting angiogenesis and branching morphogenesis	FOXO1
Napoli et al^27^	Human coronary endothelial cells	Attenuated redox‐sensitive genes (*ELK‐1* and *p‐JUN*)	Attenuation of perturbed shear stress	Increased eNOS expression
Ota et al^28^	Senescent HUVECs	H_2_O_2_‐induced premature senescence	Attenuation of premature senescence by cilostazol	Increase in Sirt1 expression
Ota et al^29^	Senescent HUVECs	H_2_O_2_‐induced premature senescence	Attenuation of premature senescence by statin (pitavastatin)	Increase in Sirt1 expression
Csiszar et al^30^	Rat carotid arteries	Cigarette smoke exposure–mediated decrease in acetylcholine response	Resveratrol	Decrease in Sirt‐mediated NK‐kB
Csiszar et al^31^	Cultured coronary arterial endothelial cells	Ad libitum diet	Caloric restriction	Attenuated TNFα‐induced ROS generation; prevented NF‐kB activation
Scalera et al^32^	Senescent HUVECs	Italian, French, and German red wines	Decreased 8‐*iso*‐prostaglandin F(2alpha) and peroxynitrite formation	Decrease in Sirt‐mediated asymmetric dimethylarginine
Ungvari et al^33^	Human coronary arterial endothelial cells	Hyperglycemia	Mitochondrial reactive oxygen species (mtROS)	Overexpression of Sirt1
Arunachalam et al^34^	HUVECs	Cigarette smoking	Reduced nitric oxide	Resveratrol‐mediated eNOS acetylation; increased NO production
Chen et al^35^	Cultured endothelial cells	Oscillatory flow	Increased Sirt1‐eNOS association and eNOS deacetylation	Enhanced NO production
Gracia‐Sancho et al^36^	HUVECs	Resveratrol	Increase in Sirt1 and mitogen‐activated protein kinase 5	Increased expression of the transcription factor Kruppel‐like factor 2
Homma et al^37^	Human adult endothelial cells, embryonic stem (ES) cells, and human iPS‐derived ECs (iPSECs)	Proliferative potential, potential for migration, and tolerance to oxidative stress	Expression of Sirt1, a nicotinamide adenine dinucleotide (NAD+)‐dependent histone deacetylase, is higher in embryonic stem cell–derived endothelial cells than in human adult endothelial cells	Higher expression of Sirt1 in iPSECs than in HAECs
Ito et al^38^	Senescent HUVECs	miR‐34a expression increases in senescent HUVECs	Overexpressing miR‐34a inhibits Sirt1 protein expression	Forced expression of Sirt1 blocks the ability of miR‐34a to induce senescence
Kao et al^39^	Cardiac coronary ECs from patients receiving CABG	Resveratrol‐induced Sirt1 activation	Sirt1 expression was decreased in aged and atherosclerotic vessels in vivo	Decreased oxidative stress by resveratrol‐induced Sirt1 activation
Stein et al^40^	Aortic rings and HAECs	Hypercholesterolemic *ApoE*^−/−^ C57Bl/6 mice	Sirt1 prevents oxidative stress, inhibits NF‐kB, and diminishes expression of ICAM‐1 and VCAM‐1	Sirt1 diminishes endothelial activation in *ApoE*^−/−^ mice
Menghini et al^41^	Senescent HUVECs, HAECs, HCAECs, atherosclerotic plaque	MiR‐217 inhibits Sirt1 expression during senescence	Antagomir of MiR‐217 partially restores senescence in ECs	MiR‐217 and Sirt1 are negatively correlated in atherosclerotic plaque
Zhao et al^42^	Bone marrow–derived EPCs	Cell cycle and apoptosis	MiR‐34a overexpression led to significantly increased EPC senescence with 40% Sirt1 reduction	miR‐34a impairs EPC‐mediated angiogenesis by induction of senescence via inhibiting Sirt1
Zu et al^43^	Endothelial cells isolated from porcine aorta	Senescence during 1 month of repetitive passages	mRNA and protein of Sirt1 were decreased; LKB1, a serine/threonine kinase, and AMPK (Thr172) were increased in senescent cells	Sirt1 promotes deacetylation, ubiquitination, and proteasome‐mediated degradation of LKB1
Guarani et al^44^	HUVECs, zebra fish, and mice	Sirt1 regulates endothelial function and angiogenesis	Sirt1 deficiency impairs endothelial growth, migration, and angiogenesis	Reversible acetylation of the Notch signaling component (NICD)
Mortuza et al^45^	Dermal‐derived human microvascular ECs; human umbilical vein ECs; bovine retinal microvascular ECs	Chemically induced activation of Sirt1 reduces oxidative stress in HG‐treated endothelial cells	High glucose decreases Sirt1‐Sirt7	Sirt1 activators reduce glucose‐induced accelerated aging through FOXO1; histone acetylase P300 and Sirt both regulate each other

HUVECs indicates human umbilical endothelial cells; eNOS, endothelial nitric oxide synthase; NO, nitric oxide; EPCs, endothelial progenitor cells; TNFα, tumor necrosis factor alpha; ROS, reactive oxygen species; BAEC, bovine aortic endothelial cells; HepG2s, human liver hepatocellular carcinoma cell line; EGF, epidermal growth factor; PAI‐1, plasminogen activator inhibitor‐1; MAPK: mitogen‐activated protein kinase; ELK‐1, ETS domain‐containing protein Elk‐1; p‐JUN, phosphorylated Jun proto‐oncogene; FOXO, forkhead box O; Mmp14, matrix metalloproteinase 14; NK‐kB, nuclear factor kappa‐light‐chain‐enhancer of activated B cells; iPS: induced pluripotent stem cell; HAEC, human aortic endothelial cells; CABG, coronary artery bypass graft; ICAM, intercellular adhesion molecule; VCAM, vascular cell adhesion molecule; HCAEC, human coronary artery endothelial cells; LKB, liver kinase B1; AMPK, 5' AMP‐activated protein kinase; HG, high glucose.

Endothelial senescence is associated with a progressive decline of eNOS function and Sirt1 expression, having Sirt1 itself a role on eNOS through deacetylation at lysines 496 and 506 in the calmodulin‐binding domain.^23^ The reduction of Sirt1 is associated with upregulation of specific microRNAs such as mir‐217 and mir‐34.^41^ Specific antagonism of mir‐217 was shown to counteract endothelial senescence in different endothelial cell lineages. Furthermore, expression of mir‐217 and Sirt1 was negatively correlated in atherosclerotic tissues, suggesting that factors increasing MiR‐217 can promote endothelial senescence. Interestingly, hyperglycemia was shown to increase MiR‐217, promoting diabetes complications.^46^ In addition, overexpression of Sirt appears to postpone the senescent phenotype of endothelial cells through Sirt‐induced epigenetic modifications of protein or through mir‐34a.^38^ Collectively, these results are consistent with the concept that Sirt1 activity plays a major role in the prevention of CVD.

We showed that insulin resistance and subclinical atherosclerosis are associated with Sirt1 downregulation in monocytes and atherosclerotic plaques^47^; in addition, glucotoxicity and lypotoxicity appear to quench Sirt1 expression in monocytic cells. The pathophysiological meaning of depressed Sirt1 expression in monocytes has been demonstrated in C57Bl/6 mice with a targeted deletion of *Sirt1* in macrophages (Lys‐Cre), which showed a metabolic syndrome–like phenotype.^48^ In subjects at risk for diabetes, downregulation of Sirt1 resulting from metabolic toxicity reduced the expression of tissue inhibitor of metalloproteinase 3 (TIMP3), a protease inhibitor with antidiabetic and antiatherosclerotic functions.^49–52^ TIMP3 exerts its functions mainly through the inhibition of ADAM‐17, also known as TNF‐alpha converting enzyme. Specifically, hyperglycemia and hyperlipidemia reduced Sirt1 activation of the TIMP3 promoter, which caused increased endothelial activation and inflammation within atherosclerotic plaques in diabetic subjects.^51^ Because soluble adhesion molecules such as VCAM‐1 and ICAM‐1 are shed by ADAM17, it is intriguing to hypothesize that a Sirt1‐TIMP3‐ADAM17 pathway is active early in the pathogenesis of endothelial dysfunction. More recently, we demonstrated that loss of TIMP3 can alter FoxO1 localization at endothelial and mesangial levels, potentially promoting dysfunctional activation of autophagy in the kidney.^53^ Because autophagy is a powerful antiaging mechanism in the kidney, we hypothesized that hyperglycemia enforces aging in the microvascular environment through the Sirt1‐TIMP3‐ADAM17 pathway. Recently, Mortuza^45^ showed that microvascular endothelial cells exposed to high glucose show evidence of early senescence. They found that high glucose induced reduction in *FOXO1* DNA‐binding ability and antioxidant target gene expression. Collectively, these data suggest that insulin resistance and hyperglycemia, by decreasing the expression of longevity‐associated genes such as *Sirt1*, predispose to reduced life expectancy over the background of genetic and environmental stressors. Other sirtuins, in addition to SIRT1, can play a role in endothelial homeostasis. Knockdown of *SIRT6* in human umbilical endothelial cells (HUVECs) increased the expression of proinflammatory cytokines, the prostaglandin system, extracellular‐matrix remodeling enzymes, the adhesion molecule ICAM‐1, cell migration, and cell adhesion to leukocytes.^54^ Cardus et al^55^ showed that *SIRT6* depletion by RNA interference in HUVECs and aortic endothelial cells reduced cell proliferation, increased the fraction of senescence‐associated β‐galactosidase‐positive cells, and diminished the ability of the cells to form tubule networks on Matrigel. Finally, Liu et al^56^ found that the pharmacologic inhibition of SIRT2 attenuates oxidant‐induced cell toxicity in endothelial cells. Collectively, these data emphasize the important protective role of sirtuins, especially SIRT1, in endothelial cells; preliminary data are emerging about a functionally important role of other sirtuins in endothelial protection.

### p66Shc, Endothelial Biology, and Metabolism

Another important mediator that is activated by altered glucose metabolism and is involved in vascular senescence is p66Shc, which operates as a redox enzyme and is linked to apoptotic cell death.^57^ Protein kinase C (PKC), which is induced by hyperglycemia, activates the mitochondrial localization of p66Shc, which in turn induces oxidative stress.^58^ In agreement with its pro‐oxidant feature, *p66Shc*'s genetic deletion increases life span in SV/129 mice by about 30%.^59^ We found that p66Shc expression is increased in peripheral blood mononuclear cells of T2DM patients compared with controls and is correlated with the degree of systemic oxidative stress.^60^ In addition, the expression of *p66Shc* is increased in the setting of experimental ED.^61^ SV/129 *p66Shc*^−/−^ mice are protected against experimental diabetic glomerulopathy, with reduction of mesangial reactive oxygen species (ROS) levels, extracellular matrix deposition, and glomerular endothelial cell apoptosis.^62^ The inhibition of p66Shc by coagulation protease‐activated protein C may exert a cytoprotective effect on diabetic nephropathy.^63^ p66Shc deletion prevents the development of diabetic cardiomyopathy by reducing cardiomyocyte death and preserving the pool of cardiac stem cells from oxidative damage.^64^ p66Shc is also involved in the mechanisms that impair diabetic wound healing: on both SC/129 and C57Bl/6 backgrounds, *p66Shc*^−/−^ diabetic mice have accelerated wound healing and do not develop the typical features of nonhealing diabetic wounds and aged skin characteristics.^65^ *p66Shc*^−*/*−^ SV/129 mice are also protected against hyperglycemia‐induced ED through reduced peroxynitrite generation and lipid peroxidation and enhanced antioxidant defenses.^66^ The mechanism is probably mediated by the ability of p66Shc to inhibit Akt signaling and eNOS phosphorylation.^67^ p66Shc appears to also exert a relevant role in terms of vascular “metabolic memory.” Paneni et al^68^ showed that in human aortic endothelial cells exposed to high glucose and aortas of diabetic SV/129 mice, activation of p66Shc by protein kinase C βII persisted after returning to normoglycemia. Deletion of p66Shc also protects from ischemia/reperfusion brain injury through blunted production of free radicals in C57Bl/6 mice.^69^ The relationship between aging, ED, and p66Shc was further explored by Francia et al,^70^ who found that *p66Shc*^−^^/^^−^ SV/129 mice showed an endothelial phenotype consistent with delayed aging. The link between p66Shc and ED is substantiated by the finding that p53 induces the expression of p66Shc, especially in response to angiotensin II, which in turn impairs endothelium‐dependent vasomotor function.^71^ In the macrovasculature, deletion of p66Shc prevents the development of early atherosclerotic lesions in SV/129 mice fed a high‐fat diet^72^ and reduces the development of advanced atherosclerosis in the *ApoE*^−^^/^^−^ mouse model on a mixed SV/129‐C57Bl/6 background.^73^ p66Shc also emerges as an important link between vascular disease and metabolism. p66Shc‐generated oxidative stress is crucial for the development of visceral fat through modulation of the insulin signal and thermoinsulation. Indeed, *p66Shc*^−^^/^^−^ mice are resistant to obesity induced by diet and leptin deficiency.^74–75^ Deletion of *p66Shc* also seems to improve insulin sensitivity in obese diabetic mice on a SV/129 or mixed background, although this effect is controversial.^76^ As deletion of p66Shc prevents insulin‐resistance, delays aging, and protects from aging‐associated diseases, one wonders why p66Shc has been selected and what its physiological role is. Giorgio et al^77^ showed that when *p66Shc*^−^^/^^−^ mice were subjected to food competition and exposed to winter temperatures while living in a large outdoor enclosure for a year, they had decreased survival compared with wild‐type hybrid C57Bl/6‐SV/129 controls. This makes *p66Shc* a candidate thrifty gene, being evolutionarily selected as advantageous for hunter‐gatherer populations, but extremely detrimental when there is constant abundance of food, contributing to the obesity and diabetes epidemics.^78^ It has been shown that p66Shc expression is regulated by Sirt1; Zhou and colleagues demonstrated that the repression of p66Shc expression by Sirt1 contributes to the protection of hyperglycemia‐induced endothelial dysfunction.^79^ Collectively, these studies have identified for the first time an intimate link of these 2 life span–determinant proteins, sirtuin and p66Shc, in the control of vascular homeostasis.

## Longevity Genes, Insulin Resistance, and Endothelial Repair

The presence of competent insulin signaling is important not only in the maintenance of endothelial function but also for endothelial regeneration.^80–81^ Repair of a damaged endothelial layer is achieved with the contribution of so‐called endothelial progenitor cells (EPCs),^82^ which participate in endothelial homeostasis and stimulate the formation of new blood vessels. Shortage of EPCs is considered a mechanism promoting cardiovascular disease development and progression.^83^ Despite some uncertainty about their definition,^83–84^ EPCs have been consistently found to be reduced in the peripheral blood of subjects with cardiovascular risk factors, especially in the presence of macroangiopathy.^81,85–86^ These abnormalities may be implicated in premature aging of the vascular system, which is characterized by a decreased capacity for neovascularization and repair.^87–88^ In this context, insulin resistance exerts additive effects on vascular regenerative capacity. Older humans experience increased bone marrow failure and poorer hematologic tolerance of cytotoxic injury. Indeed, advanced age is a major determinant of bone marrow failure and predicts a poor mobilization response after bone marrow stimulation.^89^ G‐CSF‐induced EPC mobilization is impaired in young and aged diabetic patients compared with controls, resembling an accelerated aging phenotype.^90^ A simulation suggests that a small percentage of EPCs homing to the endothelium per year could make a significant contribution to the replicative capacity of the endothelium and the prevention of senescence.^91^ Therefore, augmented risk factor–mediated endothelial injury in the absence of sufficient circulating EPCs is expected to enhance the progression of CVD. Several cellular events are associated with premature senescence in progenitor cells. Although there are limited data, Sirt1 appears to play a role in the premature aging of EPCs: mir‐34a, which was recently reported to be a tumor suppressor, targets Sirt1. Zhao and colleagues showed that cultured rat EPCs transfected with miR‐34a display significant impairment in tube‐forming activity, suggesting that miR‐34a overexpression decreased EPC angiogenic function; they also revealed that overexpression of miR‐34a significantly increased the percentage of SA‐β‐gal staining, an index of senescence.^42^ Furthermore Balestrieri et al^92^ observed that high glucose impairs the generation and function of EPCs in culture, with concurrent reduction in Sirt1 expression. Therefore, sirtuins exert an important role in mediating the longevity of progenitor cells and, indirectly, may be a potentially useful tool for stimulating endothelial repair, angiogenesis^26^ and protection of the heart against ischemic insults.^93^ Metabolic control can affect EPCs in both type 1 and type 2 diabetes.^94–95^ Again Balestrieri et al^96^ showed that the relationship between poor metabolic control and EPC number is mediated by Sirt1; they showed that Sirt1 expression is reduced via increased platelet‐activating factor receptor activation.

**Figure 1.**
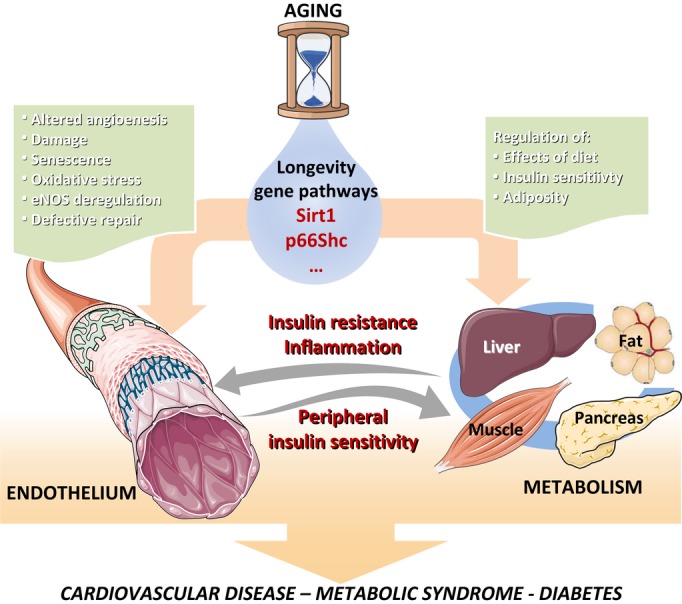
This illustration depicts the interconnections between the endothelium and metabolism in the setting of aging, which collaborate to promote cardiovascular disease, metabolic syndrome, and diabetes. eNOS indicates endothelial nitric oxide synthase.

Data indicate that p66Shc is also a molecular target to modulate endothelial repair in the setting of metabolic diseases and diabetes. Di Stefano et al^97^ found that mouse bone marrow (BM)–derived progenitor cells cultured in high glucose show higher levels of *p66Shc* gene and protein expression as well as oxidative stress than those exposed to normal glucose levels. Conversely, p66Shc‐defective BM cells were not sensitive to high glucose and developed toward the endothelial lineage. The mechanisms were related to preserved eNOS activity, reduced ROS, and accumulated nitrotyrosine. As a functional readout, *p66Shc*^−^^/^^−^ EPCs cultured from SV/129 mouse BM cells showed enhanced angiogenic potency in the Matrigel plug assay in vivo. These data indicate an intimate connection between insulin resistance, longevity genes, and endothelial biology: the network involving sirtuins and p66Shc may include other longevity pathways related to metabolic regulation^98^ (Figure 1). It is of utmost importance that the relationship between metabolism and cardiovascular aging involves stem/progenitor cells derived from the bone marrow, which is a reservoir of regenerative cells for several peripheral tissues.^80^

## Telomeres, Insulin Resistance, and Progenitor Cells

**Figure 2.**
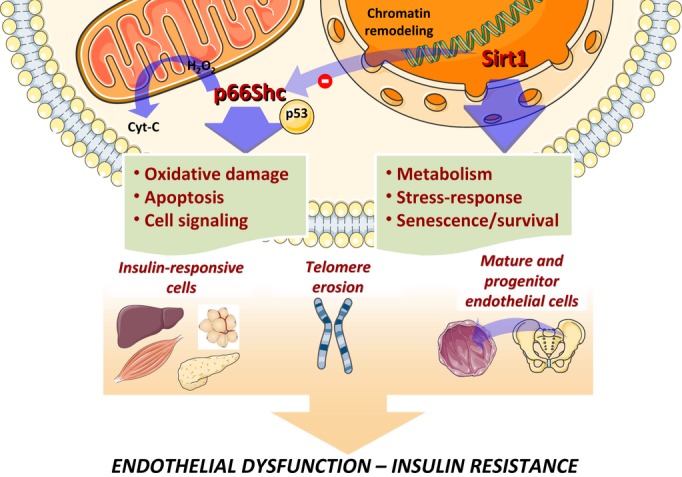
The molecular interrelationship between the longevity genes *Sirt1* and *p66Shc* in the induction of insulin resistance and endothelial dysfunction. As demonstrated by Zhou et al,^79^ *Sirt1* represses *p66Shc* transcription by chromatin remodeling, whereas P53 may be part of this molecular network as a modulator and/or downstream effect. Both reduced *Sirt1* and excess *p66Shc* expression exert negative effects on mature endothelial cells, EPCs, and insulin‐responsive cells that regulate metabolism. Along with telomere erosion, these life span–determinant mechanisms induce endothelial dysfunction and insulin resistance, which favor the aging of the cardiovascular system. EPC indicates endothelial progenitor cell.

Telomeres are specific chromatin structures at the ends of eukaryotic chromosomes that prevent the recognition of chromosomal ends as double‐stranded DNA breaks, thereby protecting these regions from recombination and degradation.^99^ Among proteins associated with telomeric DNA, telomerase and telomeric repeat binding factors 1 and 2 (TRF1, TRF2) regulate telomere length and structure.^100^ There is evidence that telomere shortening occurs in human vessels, and this may be related to age‐associated vascular changes.^101^ Telomere shortening is more prominent in coronary endothelial cells from patients with coronary heart disease compared with cells from healthy subjects.^102–103^ Insulin resistance and diabetes can also affect telomere length, although data in humans are mostly limited to leukocyte telomeres. In the Bogalusa Heart Study,^104^ the relative changes in leukocyte telomere length over 10.1 to 12.8 years were correlated with insulin resistance and changes in body mass index. In T2DM patients, the mean monocyte telomere length was significantly lower than in control subjects.^105^ In the Framingham Heart Study, leukocyte telomere length from the Offspring cohort was inversely correlated with estimates of insulin sensitivity and indexes of systemic oxidative stress.^106^ In the Cardiovascular Health Study, telomere length was inversely related to diabetes, glucose, insulin, diastolic blood pressure, carotid intima‐media thickness, and interleukin‐6.^107^ Telomere dysfunction can induce irreversible cell growth arrest (“cellular senescence”), which is controlled by tumor suppressor proteins such as p53. Minamino and his group^108^ showed that p53 expression in adipose tissue is crucially involved in the development of insulin resistance. These observations emphasize possible relationships between telomeres, insulin resistance, and the p53 tumor‐suppressor gene in the pathogenesis of cardiovascular disease; indeed, 1 cross‐sectional study showed that higher circulating p53 levels are associated with an increase in inflammatory markers, as well as increased carotid intima‐media thickness.^109^ As p53 is inhibited by Sirt1 and it activates p66Shc, studies are needed to demonstrate the concerted action of these elements on vascular homeostasis (Figure 2). Several studies have also found that telomere shortening is a critical determinant of EPC senescence,^110^ which can contribute to vascular aging.^111^ In healthy men, EPC telomere length was shown to be approximately 20% lower in the older compared with the middle‐aged and young men,^112^ and leukocyte telomere length is directly associated with circulating EPC levels in young healthy adults.^113^ The link between telomere length, EPCs, and senescence is aggravated by the coexistence of risk factor for CVD such as obesity^114^ and hypertension,^115–116^ typically observed in conditions of insulin resistance. Not all reports are unanimous in linking the senescence of EPCs to telomere length, as Zhang et al^117^ showed that tumor necrosis factor (TNF) alpha rather than telomere is implicated in EPC senescence. Interestingly, elevated TNF‐alpha is a hallmark of the proinflammatory state, which characterizes insulin resistance.^118^ Recent works have also shown important relationships between redox changes, premature vascular aging, and telomerase activity. In this context, Paneni et al^119^ showed that the lack of JunD, a member of the activated protein‐1 family of transcription factors and a major gatekeeper against oxidative stress, is associated with reduced telomerase activity, increased β‐galactosidase–positive cells, upregulation of the senescence markers p16INK4a and p53, and mitochondrial disruption. This observation is in keeping not only with the findings of Sahin and colleagues,^120^ who found that telomere dysfunction activates p53 which PGC‐1α thereby linking telomere and mitochondrial biology, but also with those of Kovalenko and coworkers,^121^ who showed that the disruption of the nuclear export signal of the catalytic component of telomerase is associated with defects in telomere maintenance and mitochondrial function. Jointly, these data suggest that telomere shortening may represent one of the mechanisms whereby insulin resistance causes oxidative stress, mitochondrial dysfunction, and vascular aging, particularly though induction of progenitor cell senescence.

## Therapeutic Implications and Conclusions

Metabolic strategies have been proposed to delay aging, beyond caloric restriction, acting on IIS pathway, sirtuins, mTOR signaling, and AMPK. Certain drugs such metformin, because of their specific mechanism of action, may create a cellular milieu that facilitates longevity.^122^ Statins may also exert potential beneficial antiaging activities. Several human progeria syndromes are caused by the accumulation of farnesylated proteins,^123^ which are targeted by the pleiotropic effects of statins.^124^ Finally, angiotensin II inhibitors may potentially be useful in prolonging life expectancy, as At‐II type I receptor (*Agtr1a*) knockout in mixed C57Bl/6‐SV/129 mice increased life span as well as the number of mitochondria, along with upregulation of nicotinamide phosphoribosyltransferase and Sirt3 expression.^125^

Insulin resistance disorders are intimately linked to both aging and ED, which is a major driver of CVD. Although CVD remains the major cause of death in Western countries, diabetes and the metabolic syndrome cause a marked shortening of life expectancy. A significant contribution to the accelerated aging process in insulin‐resistant individuals is thus attributable to endothelial senescence, dysfunction, and impaired repair. Interestingly, life span–determinant gene products, such as the sirtuins and p66Shc, have metabolic and vascular functions. It can be anticipated that strategies aimed at preserving endothelial health would turn out to be life‐span saving, as indirectly suggested by pharmacological intervention studies. Slowing endothelial senescence with a healthy lifestyle, combined with successful control of modifiable risk factors, may thus circumvent the ineluctable power of the genetic background. Targeted intervention on endothelial aging pathways is the next challenge.

## Pending Issues

- A direct role of altered expression of longevity‐related genes in predicting the development or progression of metabolic disorders is still lacking. Furthermore, it is unknown whether therapies acting on longevity‐associated pathways modify the clinical course of diabetic patients.
- A prolongevity (benevolent) condition of insulin resistance may be considered an evolutionarily conserved attempt to protect insulin‐dependent tissues from excess intracellular glucose.^126^ It is unclear whether ED has a role in mediating the protective effect of benevolent insulin resistance on longevity.
- The effect of the control of metabolic diseases such as diabetes on aging‐associated genes is unknown, as are the effects of lifestyle interventions that improve endothelial function.
